# Seizures as the first manifestation of chromosome 22q11.2 deletion syndrome in a 40-year old man: a case report

**DOI:** 10.1186/1752-1947-1-167

**Published:** 2007-12-03

**Authors:** Adriano R Tonelli, Kalyan Kosuri, Sainan Wei, Davoren Chick

**Affiliations:** 1 Department of Internal Medicine, Michigan State University, East Lansing, Michigan, USA; 2 DNA Diagnostic and Cytogenetics Laboratories, Department of Pediatrics and Human Development. Michigan State University, East Lansing, Michigan, USA

## Abstract

**Background:**

The microdeletion of chromosome 22q11.2 is the most common human deletion syndrome. It typically presents early in life and is rarely considered in adult patients. As part of the manifestations of this condition, patients can have parathyroid glandular involvement ranging from hypocalcemic hypoparathyroidism to normocalcemia with normal parathryroid hormone levels. The first manifestation of the syndrome might be seizures due to profound hypocalcemia.

**Case presentation:**

A 40-year-old man without significant past medical history presented with a new-onset generalized tonic-clonic seizure. He had no personal history of hypocalcemia or seizures. Physical examination was remarkable for short stature, hypertelorism, prominent forehead and nasal voice. His initial laboratory examination showed hypocalcemia (Calcium 5.2 mg/dl and Calcium ionized 0.69 mmol/l) with hypoparathyroidism (Parathyroid hormone intact < 2.5 pg/ml. NV: 14–72 pg/ml). Urine Calcium was 3 mg/dl on a spot and 88 mg in a 24-hour urine collection (NV: 100–300 mg/24 hs). The electrocardiogram showed a prolonged corrected QT interval. Echocardiogram, abdominal ultrasound and electroencephalogram were normal. A computer tomography of the brain showed basal ganglia calcification. The subtle physical findings and the presence of idiopathic hypoparathyroidism motivated the performance of fluorescent in situ hybridization which demonstrated a microdeletion on one of the homologs 22q11.2. The patient was treated with calcium citrate and calcitriol with good response.

**Conclusion:**

Microdeletion of chromosome 22q11.2 is among the most clinically variable syndromes, with more than 180 features associated with the deletion. It has a variable phenotypical expression, requiring a high level of awareness for its early diagnosis. Seizures, related to marked hypocalcemia due to idiopathic hypoparathyroidism, might be the presenting feature in an adult patient with this syndrome.

## Background

The microdeletion of chromosome 22q11.2 is the most common human deletion syndrome (1 in 4000 live births) [1,2]. Most deletions occur de novo, with autosomal dominant inheritance observed in 15% of cases [3]. The deletion has been identified in most patients with DiGeorge syndrome, velocardiofacial syndrome and conotruncal anomaly face syndrome. The chromosome 22q11.2 deletion syndrome is characterized by a congenital failure in the development of the derivatives of various pharyngeal arches and pouches with absent or small parathyroid glands [2]. It typically presents early in life and is rarely considered in adult patients. The classical features are conotruncal cardiac defects, dysmorphic facies, velopharyngeal insufficiency, immunodeficiency, learning disabilities and parathyroid dysfunction.

Parathyroid dysfunction in adults is generally due to acquired conditions such as autoimmune processes or injury of the parathyroid glands during surgery. Less commonly a genetic disorder like chromosome 22q11.2 deletion syndrome can be the culprit condition. In this syndrome the parathyroid glandular dysfunction ranges from hypocalcemia with hypoparathyroidism to normocalcemia with normal parathryroid hormone levels [1-3]. Various forms of hypoparathryoidism have been reported including late onset, transient, latent and recurrent [2].

## Case presentation

A 40-year-old man presented with a new-onset generalized tonic-clonic seizure. He did not have any history of hypocalcemia, seizures, psychiatric disorders or obvious cognitive impairments. Physical examination was remarkable for short stature, hypertelorism, hooded upper eyelids, prominent forehead, hypernasal speech and fullness of the nose over the bridge. The etiology of the seizures was attributed to the marked hypocalcemia noted on his initial laboratory evaluation (Calcium 5.2 mg/dl and Calcium ionized 0.69 mmol/l). Further work-up revealed hypoparathyroidism (Parathyroid hormone intact < 2.5 pg/ml. NV: 14–72 pg/ml) and a normal total 25 hydroxy-vitamin D (30 ng/ml. NV: 25–80 ng/ml). Thyroid stimulation hormone was normal. Urine Calcium was 3 mg/dl on a spot and 88 mg in a 24-hour urine collection (NV: 100–300 mg/24 hs).

**Figure 1:**
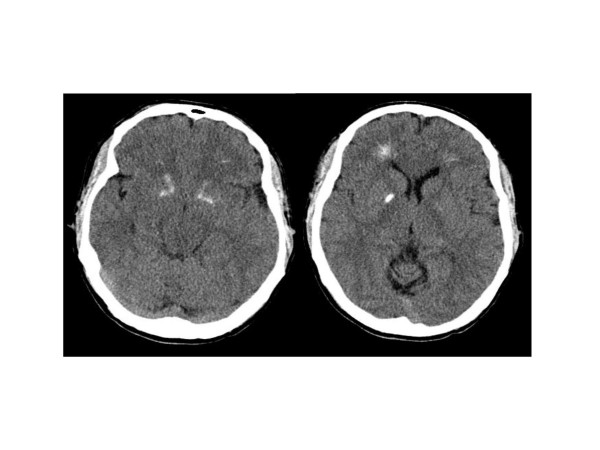
Brain computer tomography cuts of the patient, demonstrating basal ganglia and periventricular calcification.

**Figure 2:**
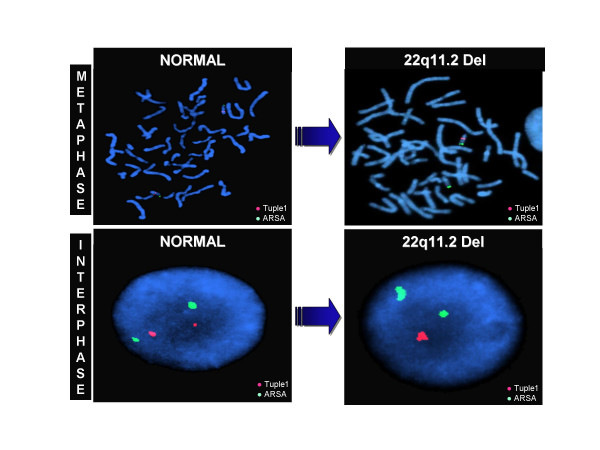
Result of FISH analysis using LSI probe (TUPLE 1) from DiGeorge/velocardiofacial syndrome critical region. TUPLE 1 (HIRA) probe was labeled in Spectrum Orange and Arylsulfatase A (ARSA) in SpectrumGreen as control. Absence of the orange signal indicates deletion of the TUPLE 1 locus at 22q11.2.

The 12-lead electrocardiogram showed a prolonged corrected QT interval. Echocardiogram, abdominal ultrasound and electroencephalogram were normal. A computer tomography of the brain showed basal ganglia calcification (figure 1). The presence of hypoparathyroidism and subtle dysmorphic facial features made chromosome 22q11.2 deletion syndrome a possible diagnosis. For this reason, fluorescent in situ hybridization was performed using DNA fluorescent probes for the DiGeorge/velocardiofacial syndrome critical region (TUPLE1) at 22q11.2 and a control probe, arylsulfatase-A (ARSA), at 22q13.3, from a commercially available source (Vysis). This is a direct-labeled dual-color probe mixture with TUPLE1 (HIRA) probe labeled in orange and ARSA probe green (figure 2). At least 50 metaphase cells and 100 interphase cells were scored for both TUPLE1 and ARSA signals. All metaphase and interphase cells analyzed show a deletion of TUPLE1 at 22q11.2 locus. The patient was treated with calcium citrate and calcitriol. He remained asymptomatic with normalization of his serum calcium level.

## Discussion

Microdeletion of chromosome 22q11.2 is among the most clinically variable syndromes, with more than 180 features associated with the deletion [1,4]. The phenotype develops over time; therefore the clinical presentation may change [4]. It has a variable phenotypic expression, with no pathognomonic or obligatory clinical features, and requires a high level of awareness for its early diagnosis [1,5]. One of the clinical features of this syndrome is seizures, which can be related to hypocalcemia, stroke, cerebellar or cerebral cortical atrophy, or transient or chronic ischemia [1,4,6-8]. A large cohort study showed that 21% of the patients with chromosome 22q11.2 deletion syndrome (62/290) had seizures, and at least 68% (42/62) of the seizures were hypocalcemic in origin [8]. There are only a few reports in the medical literature which describe new onset seizures caused by hypocalcemia in adolescence or adulthood (one report [7]), in patients not previously diagnosed with chromosome 22q11.2 deletion syndrome [1,3,7,9,10].

Some patients present with asymptomatic hypocalcemia and inappropriately low parathyroid hormone levels that leads to fluorescence in situ hybridization and the diagnosis of chromosome 22q11.2 deletion syndrome [5,7,8]. The majority of patients are diagnosed soon after birth, when the transport of calcium from mother to fetus is abruptly interrupted. The low serum calcium level generally improves over the first year of life as the parathyroid gland hypertrophies and the dietary calcium intake increase. For this reason few older patients require ongoing calcium supplementation [7,8,11].

Of note is that even when hypocalcemia is considered one of the cardinal features of the syndrome, the prevalence of this electrolyte abnormality depends on the selection criteria used, ranging from 13 to 72%. Patients with phenotypic characteristics of the DiGeorge anomaly are more likely to have clinical evidence of hypocalcemia or to have calcium levels measured. A lower prevalence of hypocalcemia is seen in clinical presentations more consistent with the velocardiofacial syndrome in which calcium levels may not be routinely checked [11]. In a large European cohort study of 558 patients known to have 22q11.2 deletions, hypocalcemia was noted in 60% of the subjects of whom 39% had presented with seizures [8].

Hypoparathyroidism can result in transient, persistent, late-onset or latent hypocalcemia. Patients who are diagnosed later in life may have a latent form of hypoparathyroidism [2,10]. There are insufficient data to describe the natural course of asymptomatic hypocalcemia diagnosed in older patients. In spite of this it is clear that in some patients, seizures may be the first manifestation of their hypocalcemia, either spontaneously or precipitated during periods of increased metabolic demand [3].

Patients with chromosome 22q11.2 deletion syndrome can have a variety of brain abnormalities when assessed by neuroimaging. Basal ganglia calcification, similar to that seen in the patient presented in this case report, have been rarely described in the literature. Possible explanations include a low incidence of this finding or a lack of computer tomography studies being performed on such patients or being performed in patients who were too young to have developed this abnormality. Most of the patients with chromosome 22q11.2 syndrome were studied with magnetic resonance imaging, possibly missing any calcification. The significance of this finding is unclear [1,10-13].

Treatment of severe symptomatic hypocalcemia requires prompt administration of parental calcium. In contrast, asymptomatic hypocalcemia may be treated with oral calcium and vitamin D supplements. Serum calcium levels should be maintained in the low-normal range to minimize hypercalciuria and the risk of development of renal calculi [11].

In summary, the diagnosis of chromosome 22q11.2 deletion syndrome during adulthood is uncommon. The condition is probably under diagnosed. It should be considered in adult patients presenting with idiopathic hypoparathyroidism. Patients with this syndrome should be informed of the symptoms that might occur with hypocalcemia, have their calcium-parathyroid hormone axis periodically checked and receive genetic counseled as there is a 50% risk of having an affected offspring [1,5].

## Conclusion

Chromosome 22q11.2 deletion syndrome has a variable phenotypic expression. This diagnosis should be considered in adult patients presenting with idiopathic hypoparathyroidism. The absence of the classical features of this condition should not exclude this pathology. Seizures, related to marked hypocalcemia, might be the initial presentation of this deletion. Treatment is relatively simple and can bring about profound clinical improvement.

## Competing interests

The author(s) declare that they have no competing interests.

## Authors' contributions

AT: Involved in drafting of the manuscript, contributions to conception and design, analysis of data;

KK: Involved in acquisition of data, conception and design. Revised the manuscript;

SW: Carried out the molecular genetic studies. Revised the manuscript;

DC: Contributed to conception and design. Revised the manuscript.

## Consent

The patient provided written informed consent for publication.
